# “…the availability of contraceptives is everywhere.”: coordinated and integrated public family planning service delivery in Rwanda

**DOI:** 10.1186/s12978-022-01325-w

**Published:** 2022-01-28

**Authors:** Adriana Scanteianu, Hilary M. Schwandt, Angel Boulware, Julia Corey, Ana Herrera, Ethan Hudler, Claudette Imbabazi, Ilia King, Jessica Linus, Innocent Manzi, Madelyn Merritt, Lyn Mezier, Abigail Miller, Haley Morris, Dieudonne Musemakweli, Uwase Musekura, Divine Mutuyimana, Chimene Ntakarutimana, Nirali Patel, Biganette-Evidente Shemeza, Gi’anna Sterling-Donaldson, Chantal Umutoni, Lyse Uwera, Madeleine Zeiler, Seth Feinberg

**Affiliations:** 1 Rutgers University, Newark, USA; 2 Western Washington University, 516 High Street MS9118, Bellingham, WA 98225 USA; 3 Spelman College, Atlanta, USA; 4 Wheaton College, Norton, USA; 5 Northwest Vista Community College, San Antonio, USA; 6 Whatcom Community College, Bellingham, USA; 7 INES, Ruhengeri, Rwanda; 8 Xavier University, New Orleans, USA; 9 University of Maryland-Baltimore County, Baltimore, USA; 10 SUNY Oswego, Oswego, USA; 11 Western Oregon University, Monmouth, USA; 12 Eastern Oregon University, La Grande, USA; 13 University of Kentucky, Lexington, USA; 14 Arcadia University, Glenside, USA; 15 Drexel University, Philadelphia, USA

**Keywords:** Rwanda, Family planning, Integration, Community health worker, Coordinated services

## Abstract

**Background:**

Contraceptive use in Rwanda tripled since 2005. This study aims to understand the role of coordinated and integrated public family planning service delivery in achieving this increase in contraceptive use in Rwanda.

**Methods:**

This qualitative study in 2018 included eight focus group discussions with family planning providers and 32 in-depth interviews with experienced family planning users.

**Results:**

Results indicate a well-coordinated public family planning service delivery system with community health workers and nurses filling different and complementary roles in meeting family planning client needs at the local level. In addition, integration of family planning into other maternal and child health services is the norm.

**Conclusions:**

The coordination and integration of family planning across both providers and services may help explain the rapid increase in Rwanda’s contraceptive use and has potential applications for enhancing family planning service delivery in other settings.

## Plain language summary

Family planning use increased from 17 to 53% in Rwanda in between 2005 and 2015. The purpose of this study is to understand the roles of two types of family planning workers in providing family planning services, how those providers work together to achieve the goal to provide public services, and how the Rwandan health system includes family planning services in a variety of other types of health services. To achieve the study purpose, 32 women with experience using modern methods of contraception were interviewed. In addition, 88 providers participated in eight group discussions to discuss these topics. The results from the interviews and group discussions showed that family planning services are easy to access for Rwandans—due to two types of family planning providers filling different roles to assist Rwandans start and keep using family planning methods. Family planning services are included in services for pregnant, delivering, and postpartum mothers—as well as services for infants and children. These are all times when those adults using the services would also be in need of family planning services. The family planning service delivery team approach—as well as including family planning services in mother’s and children’s health services likely helps explain the increase in family planning use in Rwanda. Other nations might learn from Rwanda’s service delivery approach to family planning to also increase access to family planning for their citizens.

## Background

The Alma Alta Declaration highlighted the urgent need for all persons to have access to primary care in order to meet basic human rights to health and to achieve poverty alleviation [1]. One route to increasing access to primary care for all persons, particularly for populations hardest to reach, is via community health worker (CHW) programs [2]. CHW programs provide primary comprehensive healthcare in the community [3], and extant research shows that the most effective programs have CHWs who are elected members of the communities that they serve [2]. Despite some challenges identified in sustaining CHW programs, research thus far suggests that CHWs are a worthy investment for public health [1, 4].

Health systems must be strong in order to increase access to primary health care [5]. Health systems are strengthened via effective linkage between CHWs and the next level of service, an awareness of service roles, and referral patterns [1, 6]. Unfortunately, this is not always the case, as relationships between the formal health care system and CHWs are often strained [2, 4].

CHWs are trained to provide a broad range of health services in the community, and in people’s homes. Family planning is one of those important services [7]. Family planning (FP) use reduces maternal death via a reduction in the number of unintended pregnancies and helps prevent high-risk pregnancies such as those associated with young or old age, high parity, and short inter-pregnancy intervals. Over the past two decades, FP use in developing countries has reduced the number of maternal deaths by 40%. Another 30% of maternal deaths could be averted if all contraceptive needs across the globe were met [8]. The reach of FP programs depends upon an existing well-functioning health delivery system [5]. CHW programs can extend the reach of FP programs through increasing community access—an integral component of FP program success [9]. There are some examples of CHW programs increasing FP access [7, 10]. Another route to expanding access to FP is via the integration of FP into other health services. There is accumulating evidence of successful FP integration into existing health services, including HIV care [11, 12], maternal care [13], and child health [14].

Rwanda has achieved great success in increasing family planning use: the usage rate among currently married women tripled from 17 percent in 2005 to 52 percent in 2010. In 2010, most women used injectables (15%), the pill or implants (4% each). The majority of modern method users (91%) obtain their methods from public sources [15]. For comparison, Rwanda’s neighbors have lower levels of family planning use: Burundi 29% in 2016, Uganda 39% in 2016, Democratic Republic of the Congo 20% in 2013, and Tanzania 38% in 2015 [16]. Rwanda’s government involvement is critical in the inception, design, and support of health improvements broadly in Rwanda, including family planning [17–20], as well as building health care infrastructure to reach such a wide breadth of Rwanda’s population [21]. Research shows that Rwanda’s success in improving health outcomes is due to the number, and comprehensive nature, of interventions [22].

Rwanda used inspiration from the Alma Alta Declaration of 1978 to provide primary health care to all of its citizens [23, 24]. To facilitate community ownership, and to address health care worker shortages in the wake of a four year civil war and culminating genocide, in 1995 the recently established new government of Rwanda initiated a CHW program. CHWs provide a number of health services to individuals in their communities, including FP, as the Rwandan government recognizes the role of FP use in development [17, 18, 24]. CHWs serve as the first point of contact for Rwandans seeking FP services—and they connect people with the next tier of health care provider for continued care of the client’s needs [25]. CHWs also provide FP information to clients when providing other services as a method of FP outreach. Given Rwanda’s rapid increase in FP use, this research aimed to investigate the role of coordinated and integrated FP service delivery in Rwanda from the perspective of FP providers and users.

## Methods

Data used in this research study, guided by the methodological orientation of content analysis, were collected in two districts in Rwanda in two phases in 2018. The two districts purposively selected were Musanze and Nyamasheke as they correspond to the districts with the highest and lowest rates of modern contraceptive usage in Rwanda, respectively [15]. Each district has a district hospital, health centers, health posts, and dispensaries that all provide family planning services [26]. CHWs are connected to these health facilities.

Half of the data collection activities, focus group discussions and in-depth interviews, were conducted in Musanze, while the other half were conducted in Nyamasheke. The moderators and note-takers, as well as the interviewers, used semi-structured topic guides to lead the data collection activities.

The first phase included eight focus group discussions with FP providers, four with FP nurses and four with Community Health Workers (CHW), in February 2018. Focus group discussions included eight to 12 participants, resulting in a total of 88 FP providers that participated in the study, no FP providers dropped out of the study. FP providers were purposively recruited via non-governmental organization and governmental staff intimate with the FP providers in the two districts either face to face or via the phone. Focus group discussions took place in private rooms in government buildings, with only the moderator, note-taker, and study participants present. The focus group discussion topic guide included questions about responding to client scenarios, provision of services, a risk raking exercise, and giving advice. The FP providers average age was 44, 54% were CHWs, almost all nurses were female (92%) compared to half of CHWs (52%).

The second phase of data collection in July 2018 included 32 in-depth interviews with purposively selected experienced female modern contraceptive users at least 18 years of age in the same two districts. The FP providers assisted in recruiting the experienced FP users, however, FP users were independent and not associated with the non-governmental organization or governmental staff who participated in the recruitment of FP providers. Participants for the in-depth interviews were informed of study details over the phone or in person by their FP providers, who then gauged FP users’ interest in participating in the research. None of the FP users dropped out of the study. The in-depth interviews took place inside participants’ homes or outdoors depending on the availability of private spaces and participant preferences. Only the interviewer and interviewee were present for the interviews. The topic guide included questions about personal experiences with contraceptive use, service delivery, motivations, social network influence, fears, and advice. The average age of the FP users was 38, ranging between 26 and 50. On average, users had four children, ranging between one and nine. Most were using the injectable (41%), followed by the condom (22%), and implant (19%).

Study participants signed informed consent forms in Kinyarwanda prior to participation. The male and female data collectors (CI, IM, DM, DM, BS, CU, LU) were native Kinyarwanda speaking current undergraduate students and personally familiar with Rwandan culture, thereby able to facilitate an open and culturally sensitive discussion using probes to extend the discussion beyond the interview guide questions.

Data collectors completed an in-depth training prior to data collection, including a review of study aims, research ethics, and extensive practice using topic guides to lead conversations rather than conduct surveys. After initial pilot testing, topic guides remained unchanged. Data collectors were trained to allow conversations to flow naturally and encouraged to probe into any personal experiences shared by FP providers and users. The data collectors and study participants met for the first time just prior to data collection. After introductions and cultural pleasantries, the data collectors explained the research study, their role in the study, and the role of the study participant. The focus group discussions lasted on average two hours while the in-depth interviews averaged 43 min in duration. There were no repeat data collection activities. Data collection was audio recorded, field notes were taken during the focus group discussions and after the interviews. All interviews were conducted in Kinyarwanda, then directly translated into English by native Kinyarwanda and English speakers collaboratively during transcription. Transcripts were not shared with study participants.

Data analysis was guided by content analysis [27]. Transcripts were coded by themes derived from the data using Atlas.ti 8 software [28]. First, 10 researchers coded two transcripts independently. Second, the research team then collaboratively compiled a master code list that was used to code the remaining transcripts. Group-level matrices containing quotations from study participants were further analyzed using Microsoft Excel. Results were shared back in the two districts, and capital city, of Rwanda for feedback.

Approval to conduct this study was obtained from the Institutional Review Boards at Western Washington University (EX18-030) as well from the Rwandan Ministry of Education (MINEDUC/S&T/449/2017) prior to study participant recruitment.

## Results

### Coordinated public FP service delivery

There is a high level of coordinated public FP service delivery in Rwanda. This coordination is demonstrated through the complementary roles played by different provider types, nurses and CHWs, as well as the awareness of these roles by community members (see Table 1 for organization of major, minor, and subthemes).

**Table 1:** Coordinated and integrated family planning service delivery in Rwanda themes

Major themes	Minor themes	Subthemes
Coordinated FP service delivery	First point of contact: CHW	Proximity: CHWs enable easy access to FP provider and methods
		Rapport: CHWs accessible as friends and confidants
		Provision: information, resupply, and counseling on side effects
	CHW refers client to health center	CHW may accompany client to health center
		Pregnancy tests in case of delayed resupply
		Unmanageable side effects
	Medical tests at the health center	Tests prior to initiation of new method
		Tests during sustained use
	Nurses transfer client back to CHW	Clients transferred back to CHWs for resupply of short term methods
		CHWs reconnect with all clients
Coordinated integration of FP during antenatal and postnatal care	Timing of integration	FP information during pregnancy
		FP initiation during infant health checks

#### First point of contact: CHW

Experienced contraceptive users in both Musanze and Nyamasheke agreed that the presence of CHWs has been a tremendous asset to their communities, since the presence of a CHW brings close access to a trained FP service provider and contraceptive methods. Data showed that women appreciate the improved access CHWs bring, as nearly 100% of respondents specifically raised this topic.

It is so easy to get the product that I need….It’s so easy, there are family planning providers close to home.

Female, injectables for 10 years, 50 years old, 5 children, Musanze

…I live near the community health workers so I have access to services in the way that I want and in the time that I want.

Female, injectables for 8 years, 31 years old, 3 children, Musanze

…the availability of contraceptives is everywhere…

Female, condoms, 41 years old, 5 children, Musanze

Women noted that CHWs also do home visits, sometimes even to deliver a desired contraceptive method.

Community health workers make things easier. Like, when I need a condom they bring it to my home.

Female, condom user for 2 years, 35 years old, 4 children, Nyamasheke

CHWs were not only easy to access in terms of proximity to the women’s neighborhoods, they were also accessible as friends and confidants.

The community health workers care about us. Anytime we go they give us the services we need.

Female, injectable user for 6 years, 38 years old, 6 children, Musanze

The thing that the community health workers do to make things easier is making me feel comfortable. The community health worker is the person that you already know in the village, and there isn’t anything you can be afraid of asking her because you already know each other. You can discuss anything.

Female, injectable user, 41 years, 5 children, Musanze

CHWs were noted as the first point of contact for community members interested in using family planning for information, resupply of methods, or to discuss issues they might have with method use. CHWs were the primary contacts for women due to proximity but also rapport.

Now it is easy to get contraceptives because you can find it everywhere in your village. Before the CHWs began, you had to go all the way to the health center, but now it is easier because there is someone who can give it to you in the village.

CHW, female, 50 years old, 6 children, Musanze

We’re neighbors with the community health workers, so if I have a problem I’ll go to the community health workers…

Female, condoms, 41 years old, 5 children, Musanze

The community health workers are valuable people. They are the ones who are closest with the clients. Because any problem that a client meets, they directly go to see the community health workers.

Nurse, female, 50 years old, 3 children, Nyamasheke

#### CHW refers client to health center

Women and CHWs often discussed how the CHWs would connect clients with health centers and professional providers. At the clinic, every health provider has been trained in family planning service delivery.

The other thing I can say the country helped us in is that at the health center every nurse is capable to give family planning methods. This means that they trained us about family planning. Every hospital staff knows how to give family planning methods.

Nurse, female, 35 years old, 4 children, Nyamasheke

The CHW will attend to the woman, and if the issues extend beyond the capacity of the CHW, then the CHW refers the woman to the nearest health center for assistance.

If they (CHWs) are met with a problem that is higher than their level of knowledge, they send the women to the health center.

Female, pill user for 3 years, 45 years old, 2 children, Nyamasheke

Sometimes CHWs even accompany the client to their health center appointment to increase the client’s comfort in finding the right location and to help with communication.

If I have a problem I go to the community health worker first, and if she can’t resolve my problem she can accompany me to the health center because I may be shy or don’t know where to go.

Female, condoms, 41 years old, 5 children, Musanze

Problems women faced could include the need to test for pregnancy before resupply of contraceptive methods.

…the contraceptive pills lasts between two and three months, and every three months we have to go to the provider to get more. Before they give you the pills they talk with you and ask if you are good at taking them on time, and you explain your experience. And if there is a time where you may have forgotten to take the pill, she will tell you that she can’t give you more pills yet because you may be pregnant. She will tell you to go to the health center first to make sure you aren’t pregnant and that things are okay, and then you can come back and get the pills from the nurse.

Female, pill user for 10 years, 38 years old, 3 children, Musanze

CHWs would transfer women to the health center for testing prior to initiation of contraceptive use or if the needed assistance in allevation of unmanageable side effects.

#### Medical tests at the health center

A theme present in nearly all focus groups and some interviews was the requirement for new clients to undergo medical testing at health centers prior to initiating contraceptive method use. The test results may indicate contraindication of some methods for the client. CHWs do not conduct these tests themselves, as they are only done at health centers. Rather, CHWs counsel new clients on methods, but do not initiate method use until medical tests are complete.

Even though as CHWs we teach her about different methods, we do not have the approval to give her those contraceptives. At that time we will give her a transfer to the health center. At the health center they will take some test in order to get a suitable method for her.

CHW, male, 49 years old, 4 children, Nyamasheke

The tests that providers implement may include pregnancy tests to rule out pregnancy prior to contraceptive use initiation. Providers might also test for pre-existing health conditions, such as high blood pressure, which may indicate that an individual is not eligible for certain contraceptive methods.

First, you have to go to the health centers where you get all the tests before they give you the method you want. Tests to check that you are not pregnant or that you don’t have high blood pressure and other problems.

Female, former implant user, 38 years, 5 children, Nyamasheke

Nurses at health centers also use tests to check on the health of sustained users at periodic intervals.

At health centers sometimes they test our weight to see whether after getting that method our weight is increasing or decreasing, and also our blood pressure to see whether the pressure is high so if they see that they can advise you to switch to another method. You can see that they are caring for us.

Female, pills for 4 years, 32 years old, 3 children, Musanze

#### Nurses transfer client back to CHW

Once “cleared” by the nurses to begin using contraception, women are transferred back to the CHWs for method resupply if they elected to use condoms, pills, or injectables.

…I have first to go to the health center. After getting tests and my method they give me ordinance and I give that ordinance to the community health workers as proof that I have already gone to seek out family planning so that they can continue to look after me.

Female, injectable user for 6 years, 32 years old, 2 children, Nyamasheke

CHWs also reconnect with clients after visits to the health centers to make sure they received the services they needed, discern how satisfied they are with their selected contraceptive method, and to remind the client to visit them if any issues arise.

You have to see her again after she gets back from the health center to discuss with her. I would tell her that if she has some changes in her body she should come back to see me, because this may happen.

CHW, female, 47 years old, 4 children, Musanze

Most importantly, participants were aware of what cadre of provider could help them obtain the method they desired. Participants, regardless of method used, knew that they needed to go to a health center for long term or permanent methods, while they could access short-term methods from the CHWs, only for resupply. Study participants noted the increased efficiency of the coordinated service delivery model.

Due to a lot of women that want services of family planning, there is a program that has started, some have been oriented to health centers, and others to community health workers, in order to reduce the lines of women that were waiting for the services. And also as a way to increase the services going fast for each woman that needs the services.

Female, pills for 4 years, 32 years old, 3 children, Musanze

### Coordinated integration of FP during antenatal and postnatal care

Within the system of coordinated FP service delivery, integrating FP into prenatal and postnatal care is a natural extension of the current system. CHWs and nurses teach women seeking prenatal care about FP so the women are informed about their options and have time to process the information with their husbands and others prior to birth. In some cases, women decide to initiate method use immediately after birth, and the health center is prepared to fulfill that desire. For others, women wait to initiate FP use until later in the postpartum period.

When someone is pregnant, in her 15th week of pregnancy, she has to go to the hospital to check if her baby is growing so at that time they get information…the doctor discusses with her about different contraceptive methods.

Nurse, female, 36 years old, 1 child, Nyamasheke

After they give her the information she needs, the CHW will send her to the doctor and the doctor before sending her into labor will find a method for her to use because she asked for it. He will find a method before sending her home, because she can get pregnant in those six months after birth.

CHW, female, 44 years old, 5 children, Musanze

CHWs also check on postpartum women who have not yet begun using contraceptives. This helps ensure that FP needs do not go unmet.

After giving birth, the CHW will continue to take care of her and further explain why using contraception is important…

CHW, female, 48 years old, 6 children, Nyamasheke

I first learned about family planning from nurses when I was going to get tests at the hospital during my first pregnancy. And then, I also learned about it when I had my first birth because here, when a woman gives birth, the community health workers visit her and give advice on how to use family planning.

Female, former injectable user, 32 years, 3 children, Musanze

At health centers when a woman is going to seek other services, they remind her that the family program is there, also after having her first child, the community health workers and nurses remind her that she has to choose methods in order to put space between her first child and her next children.

Female, pills for 4 years, 32 years old, 3 children, Musanze

…here in Rwanda women who are pregnant always have a CHW who checks in on how she is doing. So, in the discussion I have when I come to see how her pregnancy is, I would advise her about how to use family planning after giving birth and help her to choose the method that she wants to use.

CHW, female, 43 years old, 3 children, Musanze

#### Timing of integration

A sequence of FP integration arose often: women were informed about FP methods during pregnancy and initiated contraception during child health visits—particularly child vaccinations and weight checks. Spacing was commonly cited as the reason for FP use.

…I first understood about family planning when I was going to get a pregnancy test when I was pregnant my first time. After getting those tests, they teach us about how a woman can use family planning programs in order to help children so they aren’t close in age in order to have a healthy life and to raise children well. They try to tell us about all methods and at that time when I go back home I discussed it with my husband. After agreeing with my husband is when we decided to use family planning after having our first kid. After giving birth, like one month, I go to the kid’s vaccination and they give me that method of family planning.

Female, former implant user, 36 years, 3 children, Nyamasheke

…at that time that I came to get vaccination for my children I learned that contraception was a good thing and it helps a family in general in Rwanda, and that’s what caused me to start using contraceptives.

Female, sterilized, 40 years, 9 children, Nyamasheke

The Community Health Worker will continue the vaccines with those children and will also continue telling her about family planning.

Nurse, female, 34 years old, 2 children, Musanze

…I was going to the health center getting some tests for my first child when the nurses reminded us that the family planning program was also there...

Female, injectable user for 6 years, 32 years old, 2 children, Nyamasheke

## Discussion

Both FP providers and experienced FP users in both districts noted that public FP service delivery in Rwanda is well coordinated. CHWs in the communities and nurses at health centers occupy complementary roles to ensure easy access to contraception for women based upon their individual needs. A longitudinal study on fertility and FP use in Ghana noted that the combined efforts of FP providers and volunteers in FP program promotion resulted in the greatest fertility reduction [7]. Other researchers have also noted the important role of community based FP distribution in FP use [29]. Rwanda’s highly efficient service delivery system makes integration of FP into other services possible—such as antenatal, postpartum, and child health services, especially when the target population of the two services aligns so well. The persistent inclusion of FP information at relevant check-ups with health care professionals appears to be highly effective.

It is clear that CHWs are vital members of this efficient and integrated system. As described by the women interviewed, the CHW program in Rwanda is working well for them in terms of increasing access to information and services at their doorsteps. Research with health system administrators also highlights the integral role of CHWs in Rwanda’s health improvements [22]. CHW programs have been implemented in many different nations around the world with varying degrees of success [2]. A number of characteristics of the Rwandan CHW program arose in this research that have been considered integral to successful CHW programs in the literature: CHWs live in and are selected by their neighbors for their role, they have frequent interactions with and are well-respected by members of their communities—which serves as the most important method of supervision, and the links between CHWs and the formal health sector are strong [2, 3, 6, 30]. During the post-genocide rebuilding of the nation a monthly mandatory community service day (*umuganda*) was established that brings people together where health providers can share FP information with both urban and rural communities. The success of the Rwandan CHW program could ultimately be due to government support and respect of CHWs that sets up the pathways associated with successful CHW programs [25, 30]. Governmental support is both economical for a developing nation but may also stem from Rwanda’s progressive leadership in the global march towards gender equity. Ending gender disparities is listed as an important area of focus in *Vision 2020* (a policy planning document initiated by the Rwandan government to guide development). The nation has statutorily legislated minimum percentages of female representation in governance, and Rwanda is currently the highest in the world with 56% of the parliamentary positions held by women [31].

Since CHWs provide a range of services to their fellow community members, in addition to FP, the CHW design naturally allows for easy integration of one service into another. The “one-stop-shop” model for integration, where many different health care services can be administered at the same time and space, has been shown to increase cost efficacy in Kenya, and was generally linked to higher levels of modern method contraceptive use and knowledge among women with HIV [12]. The absence of transportation time and cost with CHWs amplifies the integration experience. In addition, the strong linkage between CHWs and the formal health system enforces the integration of FP into other health services—as does the training of all formal health system staff in FP.

This research highlights that the combination of well-coordinated service delivery and integration of FP has been critical in Rwanda’s family planning success. Yet, integration in Rwanda has not reached its full potential. Since there is a large focus on women’s fertility within marriage in Rwanda, unmarried women and adolescents are often less exposed to FP discussions and are not encouraged to use FP as strongly as married women who have already had at least one child. The Adolescent Sexual and Reproductive Health and Rights (ASRH&R) Policy of 2011 notes the lack of youth friendly facilities and emphasizes the importance of providing FP counseling and advice to adolescents. It also mentions the need for an adolescent-specific health facilities referral system [18]. Thus, integration of FP information into settings in which adolescents, unmarried, or previously married women may have more access to may be helpful in reaching these populations. Additionally, research and programs should explore the extent to which FP integration is possible for all persons years prior to a woman’s first pregnancy.

This study has a number of strengths. The qualitative methods utilized allows for deeper inquiry into the topics, and the triangulation of findings between FP providers and FP users provides an exploration of the topic from multiple perspectives. The data were collected in two districts purposively selected for having the highest and lowest contraceptive use rates in the nation as a reliability check.

Despite the study strengths, the study does suffer from a few limitations. Due to the qualitative nature of the study the findings are not generalizable. In addition, selection bias occurred due to using purposive sampling as FP providers were asked to assist in recruitment of experienced FP users and providers may have biased the sample to include more experienced and satisfied contraceptive users. The data collection might have been subject to social desirability biases due to governmental pressures or other inclinations to reflect positively on the FP program rather than provide negative information regarding availability of services or negative personal experiences. Finally, the focus of this study is limited to public sector.

## Conclusion

Rwanda’s increase in contraceptive rates over the past two decades has been astounding. While well-coordinated public FP service delivery and FP integration into general health care has been helpful in spreading awareness and usage of FP methods, the success of Rwanda’s FP program extends far beyond information integration and community-based infrastructure, and elicits continued investigation. Yet, well-coordinated FP service delivery integration, as implemented in Rwanda, introduces a viable strategy for other countries to expand FP use, and sheds light on a potential path to a promising future for FP programs throughout the world.

## Data Availability

The data generated and analyzed during the current study are available from the corresponding author on reasonable request.

## Abbreviations

CHW: Community health worker
FP: Family planning
